# Transformation of the Mental Health of the Autism Spectrum Community: Contemporary Challenges in the Post-Pandemic Era

**DOI:** 10.3390/brainsci15020178

**Published:** 2025-02-11

**Authors:** José Jesús Sánchez Amate, Antonio Luque de la Rosa

**Affiliations:** Department of Education, Universidad de Almería, 04120 Almería, Spain; jsa819@ual.es

**Keywords:** mental health, emotional regulation, autism spectrum disorder, pandemic, contemporary changes

## Abstract

**Background:** The COVID-19 pandemic represented a disruptive global event that significantly impacted mental health, posing specific challenges for vulnerable groups such as individuals with Autism Spectrum Disorder (ASD). This group faced particular difficulties due to disrupted routines, limited access to therapies, and social isolation. This study examines the changes in mental health among individuals with ASD during and after the pandemic, highlighting contemporary challenges and the mitigation strategies implemented. **Methods:** A narrative review was conducted. The search was performed in scientific databases such as PubMed, Scopus, Web of Science, PsycINFO, Teseo, Dialnet, and Google Scholar using key terms such as “ASD”, “mental health”, and “pandemic”. Studies published between 2020 and 2024 addressing the impact of COVID-19 on factors such as anxiety, depression, and stress, as well as therapeutic interventions, were selected. **Results:** Fifteen relevant studies were identified. The findings showed significant increases in the levels of anxiety and depression among individuals with ASD, which were primarily attributable to disrupted routines and social isolation. However, noteworthy innovations in virtual interventions were reported, demonstrating significant potential to mitigate the adverse effects of the pandemic. **Conclusions:** The pandemic exacerbated preexisting challenges in the mental health of individuals with ASD, revealing structural vulnerabilities in access to therapy and emotional regulation. Nevertheless, it spurred innovations in virtual interventions that could transform support for this group in the future. This analysis underscores the importance of implementing inclusive, sustainable, and adaptive policies to improve the quality of life of individuals with ASD, particularly in the context of global crises.

## 1. Introduction

The COVID-19 pandemic profoundly disrupted the daily lives of individuals with Autism Spectrum Disorder (ASD), a population that is particularly sensitive to changes in routines and structured environments. This disruption created unique challenges, as individuals with ASD faced sudden interruptions in therapies, educational activities, and adapted social spaces, significantly affecting their emotional well-being and adaptive capacities [1]. The abrupt loss of these essential supports heightened stress and anxiety, further complicating their ability to navigate an already unpredictable world [2].

ASD is characterized by a broad range of manifestations, including difficulties in social communication, repetitive behavioral patterns, and a strong reliance on daily stability. The disruption of these essential structures during the pandemic exacerbated anxiety and irritability, leading to significant challenges in maintaining previously acquired skills [3]. Many individuals with ASD struggled with the transition to virtual education and the absence of in-person therapeutic interventions, which, in some cases, led to a regression of social and cognitive development [4].

Prolonged social isolation was particularly challenging for individuals with ASD, who often depend on structured interactions to develop social skills [5]. Deprived of these environments, many exhibited an increase in repetitive behaviors and self-regulatory mechanisms, which, while temporarily beneficial, did not always support long-term social reintegration [6]. Additionally, the monotony of home life and the lack of external stimuli contributed to emotional distress and the emergence of new behavioral challenges.

For adults with ASD, the pandemic experience varied. While some found relief in reduced social pressures, others experienced setbacks due to disconnection from work and community networks, leading to increased feelings of loneliness [7]. This period highlighted the delicate balance between providing accommodations for social difficulties and ensuring meaningful social participation [6]. Ensuring access to inclusive environments that respect individuals’ needs remains crucial for long-term well-being.

Beyond its direct impact on individuals with ASD, the pandemic also altered family dynamics. The loss of external activities increased dependence on immediate caregivers, heightening stress within households and emphasizing the need for continuous external support [8]. While family environments provide stability, they can become restrictive without appropriate developmental stimuli.

Amid these challenges, digital technologies played a crucial role in maintaining therapeutic continuity [9]. Virtual therapies, previously considered supplementary, became essential during lockdowns. However, their effectiveness varied among different ASD subgroups due to sensory sensitivities, communication difficulties, and the lack of personalized approaches [10]. Some individuals benefited from the predictability of virtual sessions, while others found screen-based interactions overwhelming, limiting their participation.

Unequal access to technology also posed significant barriers. Many individuals with ASD, particularly those in vulnerable contexts, lacked adequate devices or stable internet connections, amplifying disparities in therapeutic outcomes. For digital strategies to be truly effective, they must be designed with inclusivity in mind, ensuring accessibility regardless of the socioeconomic background [6].

Another significant development was the rise of virtual support communities, which provided structured social interaction at a time when in-person options were unavailable [11]. Initially created for caregivers and professionals, these platforms also became valuable spaces for individuals with ASD to share experiences, learn from others, and maintain a sense of connection [12]. This shift highlights the potential of digital platforms not only as therapeutic tools but also as long-term mechanisms for empowerment and community integration.

As the world transitions into the post-pandemic era, individuals with ASD continue to experience the residual effects of this crisis [6]. Returning to in-person activities has been a complex process, requiring renewed strategies to address skill loss and evolving social dynamics [13]. This moment presents an opportunity to rethink support systems, ensuring they are resilient to future global disruptions.

The objective of this narrative review is to analyze the mental health changes experienced by individuals with ASD during and after the COVID-19 pandemic, highlighting the effects of routine disruption, social isolation, and therapy limitations on anxiety, stress, and emotional regulation. Additionally, this study aims to identify contemporary challenges and assess the mitigation strategies implemented to address them. By exploring these dynamics, the analysis seeks to inform the development of interventions and policies that prioritize the mental health of individuals with ASD, strengthen their resilience, and promote their long-term well-being. Within this framework, the following research questions are posed:

- What specific changes in mental health, particularly in levels of anxiety, depression, and stress, have been observed in individuals with ASD as a result of the pandemic?
- What impact have mitigation strategies, such as virtual therapies, had on supporting the mental health of individuals with ASD during the pandemic and in the post-pandemic era?
- What are the main mental health challenges faced by the ASD community in the pandemic and post-pandemic contexts, and what strategies can be implemented to ensure their well-being in future global crises?

## 2. Materials and Methods

This analysis was based on a narrative review designed to examine the transformation of the mental health of individuals with ASD during and after the COVID-19 pandemic. The primary objective was to assess the impacts of pandemic-induced conditions such as social isolation, disruption of daily routines, and limited access to therapeutic services on emotional health, anxiety, stress, and emotional regulation in this population. Furthermore, contemporary challenges in the post-pandemic period were explored, particularly regarding the adaptation of individuals with ASD to evolving social and educational environments.

Given the narrative nature of this study, it does not fall within the scope of systematic review guidelines such as PRISMA, as these are not required for this type of analysis. However, rigorous methodological steps were followed to ensure a structured and comprehensive examination of the literature.

The review also investigated the mitigation strategies implemented during and after the pandemic to counteract its adverse effects on mental health. These strategies have contributed to the transformation of intervention and emotional support approaches within the ASD community. Particular emphasis was placed on how individuals with ASD have navigated new emotional and social challenges in the post-pandemic era and how they have adapted to the ongoing changes in their daily lives.

For this analysis, well-established scientific databases, including Scopus, Dialnet, and Web of Science (WoS), were utilized due to their academic prestige and relevance to educational and health research. The process commenced with an exhaustive search within these databases, followed by the collection and selection of pertinent articles. Subsequently, a detailed analysis was conducted, extracting essential information from each study, such as authorship, objectives, methodology, participants, and findings. This information was then compared with the existing literature to evaluate the coherence and validity of the results.

This methodological approach provided a comprehensive understanding of the pandemic’s effects on the mental health of individuals with ASD, highlighting transformations of their emotional and social well-being. Additionally, it established a strong foundation for future research and the development of interventions aimed at fostering resilience and promoting the well-being of this population in the post-pandemic era.

### 2.1. Search Procedures

This narrative review followed a comprehensive bibliographic search strategy aimed at understanding the post-pandemic impacts on the mental health of individuals with ASD. During the months of October, November, and December 2024, an exhaustive search was conducted in the databases Web of Science (WoS), Dialnet, and Scopus using a combination of the following descriptors: “ASD and mental health”, “mental health and ASD post-pandemic”, “coping strategies in ASD”, “emotional challenges and ASD”, “emotional impact ASD pandemic”, and “ASD and emotional health”.

The initial search was restricted to open-access documents, specifically those categorized in the fields of education/psychology and related to the emotional and mental impacts of the post-pandemic period. Articles were included if written in English or Spanish. This initial process resulted in 198 articles. After removing duplicates, the number of articles was reduced to 111 (WoS: 9; Dialnet: 87; and Scopus: 15).

In the first selection phase, the titles and abstracts of the 111 articles were reviewed, applying the inclusion criteria (A), (B), (C), and (D) described in the following section (Table 1). From this phase, 37 articles were selected for further review. These articles were independently evaluated by two authors to confirm their inclusion based on the defined criteria. The initial agreement between reviewers after the full review was 98.81%, and discrepancies were resolved through discussion and consensus, achieving 100% agreement.

**Figure 1:**
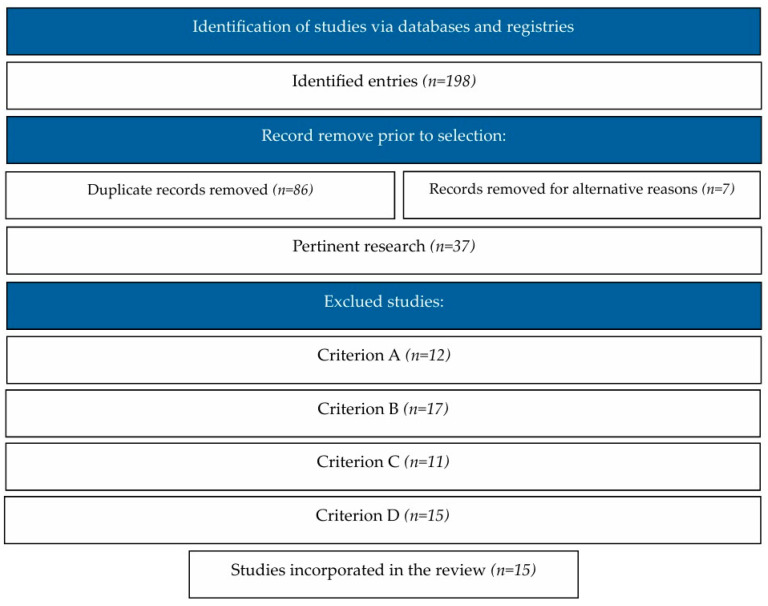
Flowchart.

The search and selection process are illustrated in Figure 1, presented as a flowchart of the study selection process.

### 2.2. Selected Search

Finally, this narrative review included 15 articles (Table 2), which were analyzed from two key perspectives: the descriptive information about the studies and their findings, and the evaluation of the studies’ quality and the validity of the data provided based on inter-rater reliability. These analyses addressed various research objectives, focusing on the emotional and psychological impacts of the pandemic on individuals with Autism Spectrum Disorder (ASD), as well as post-pandemic challenges and the coping strategies implemented to improve their mental well-being.

Firstly, attention was exclusively directed toward articles, as they offer more detailed and synthesized research on the topic. Studies published in both English and Spanish were included, although the majority of relevant articles identified were in Spanish. Additionally, the publication period was limited to between 2020 and 2024 to ensure the inclusion of relevant documents on the impact of the pandemic on the mental health of individuals with ASD and the mitigation strategies implemented during and after the lockdown period. Finally, given the focus on mental health and contemporary post-pandemic challenges, the search was restricted to publications within the fields of psychological, educational, and health research, with an emphasis on studies addressing emotional well-being and the difficulties stemming from social isolation and disrupted routines.

## 3. Results

The COVID-19 pandemic generated profound transformations of the mental health and emotional well-being of individuals with Autism Spectrum Disorder (ASD), presenting challenges that remain relevant in the current context. Moreira et al. [14] highlighted how therapeutic strategies implemented during the pandemic, such as cognitive–behavioral therapy and the use of digital tools, became essential in managing stress and anxiety while maintaining emotional stability. These practices not only were crucial during the periods of strict confinement but also served as adaptive models in the post-pandemic period. Similarly, Valdez et al. [15] pointed out how social isolation and restrictions led to increases in emotional disorders and regressions of previously acquired skills, underscoring the need for sustainable strategies to reduce emotional vulnerability. Likewise, Amorim et al. [17] analyzed how the disruption of routines had a lasting impact on stress management and emotional adaptation, emphasizing the role of structured activities in fostering resilience.

From a behavioral and psychopathological perspective, the effects of confinement highlighted the need to adapt interventions to the current challenges. De la Cruz [18] observed that anxiety, irritability, and repetitive behaviors, which increased during the pandemic, persist today, stressing the importance of strengthening caregiver support to manage these patterns effectively. Additionally, Soubeste et al. [19] identified that disruptive behaviors and aggression, exacerbated by the interruption of therapies and reduced social interactions, continue to impact the social and emotional dynamics of individuals with ASD. Similarly, Zhao et al. [22] explored the relationship between autistic traits and the risk of developing post-traumatic stress disorder (PTSD) symptoms, highlighting how these experiences reinforce the need for therapeutic approaches that simultaneously address emotional and behavioral aspects.

The abrupt changes in routines and social isolation not only caused immediate tensions but also left lasting effects on emotional regulation and behavior. Mosquera et al. [24] noted that while some factors, such as reduced social demands, might have had a mitigating effect, prolonged isolation increased emotional vulnerability and feelings of social disconnection—issues that must still be addressed in the post-pandemic period. Lugo et al. [25] emphasized how the lack of social interaction heightened episodes of anxiety and altered the perception of daily activities, underlining the importance of reestablishing support networks to facilitate a transition toward more balanced functioning. Similarly, Núñez et al. [26] observed that the behavioral and emotional changes caused by confinement heightened tension in family dynamics, emphasizing the need for structured strategies to restore emotional and behavioral equilibrium.

In response to these challenges, the coping strategies developed during the pandemic have evolved to meet current needs in a context that demands flexibility and continuous adaptation. Prieto et al. [21] highlighted how family interventions and personalized therapies remain essential for mitigating emotional stress and promoting psychological well-being among individuals with ASD. These practices not only address immediate challenges but also provide a resilient model for the future. Similarly, Coelho et al. [16] pointed out that family coexistence during periods of confinement led to unexpected benefits, such as improved communication and autonomy, which can be integrated into long-term strategies to strengthen social and emotional relationships in the post-pandemic context.

The psychological and behavioral impacts of the pandemic remain a priority in designing interventions that respond to the social and emotional transformations experienced by the ASD community. Levante et al. [27] noted that challenging behaviors and elevated anxiety levels, which intensified during confinement, continue to pose obstacles in the process of social and emotional integration. Martínez et al. [28] compared the experiences of individuals with ASD and neurotypical individuals, highlighting how the former faced significantly greater emotional deterioration, reinforcing the need for specialized approaches in the current context. Vibert et al. [23] emphasized that the continuity of therapeutic services played a crucial role in stabilizing emotional and behavioral symptoms during and after the pandemic, serving as a guide for future crisis scenarios. Finally, Stadheim et al. [20] observed that social regressions and increased disruptive behaviors during confinement continue to hinder the integration of individuals with ASD into their environments, underscoring the need to maintain structures and supports that promote a successful transition to normalcy.

To support and validate these findings, an evidence map is included (Table 3), gathering the studies mentioned and providing a comprehensive overview of the key factors influencing the mental health of the ASD community. This map not only illustrates the psychological and behavioral impacts of the pandemic but also highlights the strategies required to foster resilience and emotional well-being in a context that demands the continuous transformation of interventions.

## 4. Discussion

This narrative review of studies of the well-being of individuals with Autism Spectrum Disorder (ASD) reveals a multifaceted picture of the challenges they faced, both during and after the COVID-19 pandemic. The analyzed results address critical aspects such as mental health, behavioral impacts, coping strategies, and the importance of family dynamics, providing a comprehensive view of how the pandemic’s effects continue to reverberate in individuals with ASD. This discussion aims to integrate and compare the results to offer a coherent understanding of how these interconnected factors persistently affect this population.

The findings show considerable variability in the mental health and emotional well-being of individuals with ASD. The evidence suggests that the pandemic intensified emotional and behavioral difficulties, and despite efforts to mitigate these effects, many individuals with ASD continue to face significant challenges. Moreira et al. [14] documented considerable increases in anxiety, stress, and behavioral regressions in children with ASD—issues that have not disappeared entirely despite the return to pre-pandemic conditions. This phenomenon reflects the complex interaction between the immediate impact of the pandemic and the long-term consequences of disrupted routines and loss of access to continuous and specialized therapies.

On the other hand, Valdez et al. [15] and Amorim et al. [17] agree that social isolation and changes in daily structure significantly affected emotional well-being, contributing to emotional distress that still persists. The loss of social and therapeutic activities has left a lasting mark on emotional health, exacerbating the levels of anxiety and irritability. These studies emphasize that although some effects have been mitigated by re-establishing routines, emotional adaptation remains a challenge, especially when therapeutic interventions have not been adequately adjusted to meet the evolving needs of this population. This finding highlights the need for continuous therapeutic modifications to address the long-term aftermath of the pandemic.

Regarding behavioral and psychopathological impacts, studies by Soubeste et al. [19] and Stadheim et al. [20] show how the interruption of in-person therapies and the increase in social isolation led to a rise in disruptive behaviors such as aggression and irritability—effects that continue to pose challenges today. While the return to normalcy has allowed for an improvement in some cases, these behavioral alterations still require specialized attention. Furthermore, Zhao et al. [22] warn about prolonged emotional vulnerability, particularly in individuals with more pronounced autistic traits, who remain at higher risk of developing post-traumatic stress disorder (PTSD) symptoms due to the health crisis. This reinforces the idea that the pandemic not only had an immediate impact but also left emotional consequences that require long-term intervention approaches.

Moreover, the difficulty in adapting to structured routines remains evident. Coelho et al. [16] and Lugo et al. [25] documented how the lack of social interaction and the interruption of daily activities led to emotional setbacks, making it harder for individuals with ASD to fully adjust to new environments. These challenges underline the importance of restoring consistency in daily life and ensuring adequate therapeutic support tailored to the current needs. Mosquera et al. [24] emphasize how loneliness and emotional vulnerability continue to exacerbate mental health issues in adults with ASD, underscoring the urgent need to maintain consistent support networks and structured activities to foster long-term emotional and social well-being.

Regarding coping strategies, these approaches remain fundamental for managing emotional and behavioral effects in the post-pandemic context. Valdez et al. [15] and Coelho et al. [16] highlight that telematic interventions and the strengthening of family bonds were key in reducing emotional stress and helping families maintain an emotionally stable environment. However, these strategies remain necessary to sustain the well-being of individuals with ASD, who continue to face difficulties in adapting to new routines. Proactive coping strategies, such as social support and a positive reappraisal of situations, have continued to demonstrate effectiveness in mitigating post-pandemic stress.

Although the reviewed studies provide valuable insights into the post-pandemic challenges faced by individuals with ASD, it is important to acknowledge certain limitations. The methodological heterogeneity of the studies, with varying sample sizes and assessment tools, complicates direct comparisons and the generalization of the findings. Additionally, most research relied on cross-sectional data, limiting the ability to observe long-term trends in the well-being of this population. The absence of extensive longitudinal studies restricts a deeper understanding of the sustained impact of the pandemic and the effectiveness of different intervention strategies over time. Furthermore, the available studies may not fully represent diverse socioeconomic and cultural contexts, which could influence both access to interventions and the effectiveness of coping mechanisms. Addressing these gaps in future research will be essential to developing more tailored and sustainable support strategies for individuals with ASD and their families.

In summary, despite these limitations, the findings underscore the importance of adopting a comprehensive approach to address the well-being of individuals with ASD in the post-pandemic era. The combination of adaptive coping strategies, strengthening family bonds, and maintaining structured routines is crucial for improving quality of life and reducing emotional overload. Integrating these approaches can provide a solid foundation for developing more specific and effective support strategies, laying the groundwork for better stress management and improved well-being for both individuals with ASD and their families in the future.

## 5. Limitations and Perspectives

One of the main limitations of this study lies in the open access to the available scientific literature. Although a rigorous bibliographic review was conducted to ensure a solid and well-founded approach, it is possible that some relevant studies were not included due to access restrictions to certain sources. This situation, which is inherent to the availability of publications in academic and scientific environments, may have led to the unintentional omission of previous studies addressing the same topic from complementary perspectives. The existence of barriers to knowledge access represents a challenge for research, as it limits the possibility of comparing and enriching the analysis with a broader range of previous findings, highlighting the need to continue advancing towards more accessible and equitable publishing models.

## 6. Conclusions

In conclusion, the findings from the review of studies of the mental health of individuals with Autism Spectrum Disorder (ASD) during and after the COVID-19 pandemic highlight various specific changes in their emotional well-being.

First, regarding the question of what specific changes in mental health, particularly anxiety, depression, and stress levels, were observed in individuals with ASD as a result of the pandemic, the evidence shows that the pandemic significantly exacerbated depression, stress, and particularly anxiety in individuals with ASD, especially in younger subjects. During the lockdown, individuals with ASD experienced notable increases in anxiety and stress, which were accompanied by regressions of previously acquired skills, affecting their ability to socialize, handle daily situations, and develop new abilities. Although the return to routines has mitigated some effects, many of the emotional and behavioral problems observed during the health crisis persist, highlighting the need for sustained interventions and continued emotional support to address these long-term challenges.

Regarding the impact of mitigation strategies, particularly virtual therapies, on supporting the mental health of individuals with ASD during the pandemic and in the post-pandemic period, virtual therapies were a key tool in providing emotional and psychological support to individuals with ASD during the pandemic. These therapies, although not a perfect solution or a complete replacement for in-person interventions, were able to maintain the continuity of treatments, which helped reduce emotional stress in individuals with ASD. However, the effectiveness of these interventions was limited by issues such as inconsistent access to technology and technological barriers for some families, underscoring the need to ensure equitable access to these tools in the future. In the post-pandemic period, although most in-person services have become available again, virtual therapies continue to be essential as a complement to in-person interventions, providing flexibility and continuity of treatment.

Finally, addressing the questions of what the main mental health challenges are for the ASD community in the context of the pandemic and post-pandemic period, and what strategies can be implemented to ensure their well-being in future global crises, the main challenges include difficulty adapting to new routines, the persistence of emotional symptoms such as anxiety and irritability, and family stress due to the disruption of therapies and social isolation. These challenges continue to be relevant in the post-pandemic period, underscoring the urgent need to develop therapeutic approaches that integrate adaptive coping strategies, such as strengthening emotional regulation skills, reinforcing family bonds, and re-establishing structured routines. Moreover, it is crucial to design interventions that not only address the emotional and behavioral needs of individuals with ASD but also consider the overall well-being of their families, providing continuous psychological support and fostering a supportive environment at home. For future global crises, it is essential to create comprehensive and sustainable strategies that prioritize both the emotional well-being and mental health of individuals with ASD, ensuring they can better adapt to social changes and stressful situations.

In summary, the COVID-19 pandemic has had a lasting impact on the mental health of individuals with ASD, revealing the need for ongoing interventions and long-term emotional support strategies. Implementing approaches that integrate adaptive strategies for stress management and strengthening family support will be crucial to ensuring better emotional well-being in the post-pandemic context and preparing for future global crises.

## Figures and Tables

**Table 1:** Selection criteria.

	Inclusion Criteria	Exclusion Criteria
A	Empirical studies or research published in peer-reviewed scientific journals in English or Spanish	Other publications (documents with theoretical content or related to non-educational aspects, books, etc.)
B	Open access through the internet	No open access through the internet
C	Categories restricted to “Research in Psychology/education” related to the proposed objectives	Documents related to other aspects not included in the objectives of this study
D	Specific documents for people with ASD	Studies or experiences involving other populations

**Table 2:** Description of the included studies.

Author	Year	Objective	Participants	Methodology	Results
Moreira, Alarcon, and Gutiérrez [14]	2022	Analyze how clinical psychologists contributed to the mental health care and emotional support of children with Autism Spectrum Disorder (ASD) during the COVID-19 pandemic, focusing on the strategies implemented to mitigate mental health challenges in this vulnerable population.	15	Qualitative and Quantitative	The COVID-19 pandemic lockdown significantly impacted the mental health of children with Autism Spectrum Disorder (ASD). Regressions in previously acquired skills and increased anxiety, stress, and disruptive behaviors such as irritability and self-injurious actions were observed. Additionally, the interruption of therapeutic care intensified emotional vulnerability, affecting their mental stability and socialization abilities. Clinical psychologists played a fundamental role by applying psychoeducational strategies directed at parents to manage the behavioral and emotional changes in children. They implemented personalized interventions, including sensory stimulation, cognitive–behavioral therapy, and the use of digital tools to maintain connection with patients. Furthermore, they promoted the recovery of comprehensive care and the readaptation of routines, which were crucial to stabilizing emotional well-being in this critical context.
Valdez, Montiel, Cristiane, Rattazzi, Rosoli, Barrios, Cukier, García, Manrique, Liz, Lima, Amigo, Besio, and Garrido [15]	2021	Investigate how the pandemic and social isolation impacted the emotional health of individuals with Autism Spectrum Disorder (ASD) and their families, analyzing changes in behavior, mood, sleep, and eating patterns, as well as the repercussions of disrupted therapeutic and educational services.	1826	Qualitative and Quantitative	The emotional health of individuals with Autism Spectrum Disorder (ASD) was significantly affected during the pandemic. Reports indicated increases in anxiety (63%), irritability (64.9%), and mood disturbances, while more than 57% of families observed emotional and behavioral regressions. Disruptions in therapeutic services heightened family stress; however, telematic interventions provided partial support, reducing the stress associated with travel and encouraging caregiver involvement in managing their children’s emotional well-being. Additionally, outdoor activities and walks had positive effects on emotional regulation, emphasizing the importance of incorporating outdoor activities as a strategy to improve emotional well-being during confinement contexts. These findings highlight the need to strengthen therapeutic models that prioritize the emotional health of individuals with ASD and their families, particularly during crisis situations.
Coelho, Medeiros, Gálvez, Núñez, Le Royd, Riquelme, and López [16]	2022	Explore how the COVID-19 pandemic lockdown affected the mental health of children with Autism Spectrum Disorder (ASD) from the perspective of their parents, identifying both emotional challenges and factors that promoted resilience within the family context.	118	Qualitative	The pandemic and lockdown had significant repercussions on the mental health of children with Autism Spectrum Disorder (ASD), as evidenced by increases in emotional dysregulation, anxiety, and disruptive behaviors such as irritability and aggression. However, benefits were also observed, including strengthened family bonds and improvements in communication and autonomy, fostered by shared time at home. Parents played a key role in managing their children’s emotional well-being, highlighting the need to integrate psycho-emotional support strategies into therapeutic interventions. Despite the challenges, parental empowerment and family time emerged as fundamental tools for emotional stabilization, underscoring their inclusion in therapeutic intervention models in the post-pandemic period.
Amorim, Catarino, Miragaia, Ferreras, Viana, and Gurdiano [17]	2020	Understand how children with Autism Spectrum Disorder (ASD) and their families experienced social isolation during the COVID-19 pandemic, evaluating changes in mental health, behavior, emotional management, and anxiety levels compared to children without neurodevelopmental disorders.	99	Qualitative and Quantitative	During the lockdown, the mental health of children with Autism Spectrum Disorder (ASD) and their caregivers was profoundly affected, with increases in anxiety and emotional difficulties. Children exhibited behaviors such as irritability and obsessions, while caregivers experienced elevated levels of anxiety, reflecting a significant emotional burden. The absence of routines exacerbated anxiety and complicated children’s adaptation to the confinement. These findings highlight the need to adopt strategies that promote emotional management and the maintenance of routines to enhance the well-being of the ASD community and their families in the post-pandemic stage
De la Cruz [18]	2022	Analyze the emotional and psychopathological impact of the COVID-19 pandemic lockdown on individuals with Autism Spectrum Disorder (ASD) and/or disabilities, evaluating changes in their mental health during confinement and their recovery in the “new normal”, as well as their relationship with family and social dynamics.	35	Quantitative	The lockdown had a significant impact on the mental health of children with Autism Spectrum Disorder (ASD), as evidenced by increased anxiety, irritability, and repetitive behaviors. While these symptoms decreased with the transition to the new normal, some, such as apathy, persisted. Initially, family relationships and learning were negatively affected but showed significant improvements as structured routines were reestablished. The role of caregivers was crucial: those with greater adaptability were more effective at mitigating the emotional impact on children. These findings highlight the need for interventions aimed at strengthening the emotional health of families and facilitating their adaptation in the post-pandemic stage.
Soubeste, Salomon, and Sadaniowski [19]	2023	Study the impact of mandatory preventive social isolation (ASPO) on the emotional and behavioral health of children with Autism Spectrum Disorder (ASD) and their families, focusing on access to therapies, educational continuity, and family strategies implemented during the lockdown.	12	Qualitative and Quantitative	The ASPO significantly impacted the mental health of children with Autism Spectrum Disorder (ASD), increasing anxiety, aggression, and disruptive behaviors due to the interruption of in-person therapies and limited socialization. However, shared family time strengthened bonds and facilitated progress in communication and language, highlighting the crucial roles of parents in emotional and educational management. Virtual therapies provided partial support, though access barriers persisted for many. These findings underscore the need to integrate adapted therapeutic and educational strategies that enhance emotional well-being and resilience for families in post-pandemic scenarios
Stadheim, Johns, Mitchell, Smith, Braden, and Matthews [20]	2022	Understand the impact of the COVID-19 pandemic on the mental health of children and adolescents with Autism Spectrum Disorder (ASD) and their families, evaluating the emotional and behavioral challenges resulting from social isolation and the loss of routines, as well as identifying key factors that promoted resilience.	122	Qualitative	The impact of the pandemic on children with Autism Spectrum Disorder (ASD) included regressions of social and communication skills, increases in challenging behaviors such as aggression and irritability, and mood changes, with higher levels of anxiety and sadness. Caregivers faced elevated stress due to the loss of professional support and the need to take on multiple roles. However, protective factors such as strengthened family bonds and the development of autonomy skills in some children were identified. These findings highlight the importance of integrating adapted emotional and educational supports into therapeutic interventions to address the challenges of the post-pandemic period.
Prieto, Martínez, Criado, and Martinez [21]	2022	Investigate the behavioral, emotional, social, and communicative impacts of the COVID-19 pandemic on children and adolescents with Autism Spectrum Disorder (ASD), as well as the repercussions on their families and the perceived needs during and after confinement.	140	Qualitative and Quantitative	The pandemic profoundly impacted the mental health of children and adolescents with Autism Spectrum Disorder (ASD), manifesting in high levels of stress, anxiety, and irritability. During confinement, regressions of social and emotional skills were observed, along with difficulties in emotional regulation. Safety measures, such as mask-wearing and social distancing, added complexity to the emotional well-being of this population. Families also experienced increased stress due to the interruption of therapies and the emotional burden of assuming multiple roles. These findings highlight the importance of designing interventions that prioritize the mental health of both children with ASD and their families, strengthening support networks and providing adapted resources in post-pandemic contexts.
Zhao, Li, Li, and Shi [22]	2021	Investigate the relationship between autistic traits (ATs), COVID-19-related post-traumatic stress disorder (PTSD) symptoms, and the role of anxiety sensitivity (AS) as a mediating factor, while also considering sex differences.	442	Qualitative	The study demonstrated that individuals with elevated autistic traits are more vulnerable to developing COVID-19-related PTSD symptoms, particularly women. Anxiety sensitivity acts as a key mediating factor, exacerbating PTSD symptoms in this group. The results highlight that among those with elevated autistic traits, the levels of hypervigilance are significantly higher in women. These findings suggest the need for personalized intervention approaches that include strategies to reduce anxiety sensitivity, such as cognitive–behavioral therapy, and emphasize the importance of a gender-adapted approach to post-pandemic mental health care.
Vibert, Segura, Gallagher, Georgiades, Pervanidou, Thurm, Alexander, Anagnostou, Aoki, Birken, Bishop, Boi, Bravaccio, Brentani, Canevini, Carta, Charach, Costantino, Cost, Cravo, Crosbie, Davico, Donno, Fujino, Gabellone, Geyer, Hirota, Kanne, Kawashima, Kelley, Kim, Kim, Kim, Korczak, Lai, Margari, Marzulli, Masi, Mazzone, McGrath, Monga, Morosini, Nakajima, Narzisi, Nicolson, Nikolaidis, Noda, Nowell, Polizzi, Portolese, Riccio, Saito, Schwartz, Simhal, Siracusano, Sotgiu, Stroud, Sumiya, Tachibana, Takahashi, Takahashi, Tamon, Tancredi, Vitiello, Zuddas, Leventhal, Merikangas, Milham, and Di Martín [23]	2023	Evaluate changes in symptoms and access to services during the COVID-19 pandemic in individuals with Autism Spectrum Disorder (ASD) and other neurodevelopmental disorders (NDDs), with a particular focus on the impacts on their emotional and mental well-being.	1275	Quantitative	The study identified four subgroups of youth with Autism Spectrum Disorder (ASD) and neurodevelopmental disorders (NDDs) based on changes in symptoms and services: widespread symptom deterioration (20%), services mostly modified (23%), services mostly lost (6%), and average changes in symptoms and services (53%). It was observed that the worsening of symptoms, accompanied by increased anxiety and stress, was related to concerns about COVID-19 and lifestyle stress. On the other hand, the continuity of services, even in modified formats, contributed to greater emotional and behavioral stability. These findings highlight the need to ensure adapted support services, prioritizing mental well-being in youth with ASD/NDDs during crisis situations.
Mosquera, Mandy, Pavlopoulou, and Dimitriou [24]	2021	Explore how autistic adults manage social expectations before and during the COVID-19 lockdown in Spain, highlighting the impact on their emotional well-being and the mental health challenges they faced.	10	Qualitative	The lockdown reduced social interactions, decreasing the pressure of “social masking”, a strategy that had previously generated high levels of stress and anxiety in autistic adults. However, this isolation exacerbated feelings of loneliness, vulnerability, and social disapproval, negatively affecting their mental health. The lack of adequate support networks during the lockdown worsened these effects, increasing the need for interventions that address emotional and social barriers to improve the mental well-being of autistic individuals in crisis situations. These findings highlight the importance of strategies focused on mental health and the strengthening of inclusive communities.
Lugo, Gisbert, Setien, Español, Ibáñez, Forner, Arteaga, Soriano, Duque, and Ramos [25]	2021	Evaluate the psychological impact of the confinement due to the social emergency caused by COVID-19 on individuals with Autism Spectrum Disorder (ASD) (children, adolescents, and adults), analyzing changes in psychopathological symptoms, the subjective perception of changes in daily functioning areas, and stress levels.	74	Qualitative and Quantitative	During the COVID-19 lockdown, adults with Autism Spectrum Disorder (ASD) showed a significant improvement in their emotional well-being, with a reduction in psychopathological symptoms and lower stress levels. In contrast, children and adolescents with ASD did not experience significant changes in symptoms, except in areas such as anxiety and somatization, which increased significantly. Caregivers, on the other hand, reported an increase in stress. While improvements in food quality and a reduction in the initiation of social interactions were observed, it was noted that the reduction in social demands benefited some individuals with ASD, but it increased the emotional burden on caregivers.
Nuñez, Le Roy, Coelho, and López [26]	2021	Evaluate the impact of the COVID-19 pandemic outbreak on the behavior of individuals with Autism Spectrum Disorder (ASD), identifying predictive factors that contributed to the increases in behavioral difficulties and mental health challenges.	270	Qualitative and Quantitative	A total of 45% of parents reported an increase in the intensity and frequency of their children’s behavioral difficulties during the lockdown, with their mental health being directly affected as well. These findings suggest that psychosocial factors, such as family stress and the mental health of caregivers, played a crucial role in the behavior of children with Autism Spectrum Disorder (ASD) during the confinement period.
Levante, Petrocchi, Bianco, Castelli, Colombi, Keller, Narzisi, Masi, and Lecciso [27]	2021	Examine the psychological impact of the COVID-19 outbreak on the families of children with Autism Spectrum Disorder (ASD) and typically developing children, comparing emotional responses and behavioral issues during the lockdown.	120	Qualitative and Quantitative	During the lockdown, parents of children with Autism Spectrum Disorder (ASD) reported higher levels of distress and more behavioral problems compared to families of typically developing children. Children with ASD displayed more positive emotions but engaged in fewer play activities compared to their typically developing peers. Additionally, parents of children with ASD reported that their children experienced higher levels of negative emotions. Parental stress was a significant factor affecting children’s emotional responses and adaptive behavior, such as play activities. These findings highlight the need for interventions that address parental stress and improve the emotional well-being of both children and caregivers in crisis situations like the pandemic.
Martínez, Moreno, and Piqueras [28]	2021	Analyze the differences in emotional states between a group of individuals with Autism Spectrum Disorder (ASD) and a neurotypical group before and during the COVID-19 lockdown.	214	Quantitative	During the lockdown, individuals with Autism Spectrum Disorder (ASD) exhibited significantly higher levels of aggression, irritability, hyperactivity, impulsivity, inattention, anxiety, and anticipatory anxiety compared to the neurotypical group. Additionally, autistic symptoms, including repetitive, restrictive, and stereotyped behaviors, significantly increased in the ASD group during the lockdown compared to individuals assessed before the pandemic. These findings suggest that the lockdown exacerbated the emotional and behavioral symptoms characteristic of ASD, highlighting the need for intervention strategies to mitigate the psychological impact in future crises.

**Table 3:** Evidence map.

Category	Reference	Year	Method	Key Findings
Impacts on Mental Health and Emotional Well-being	Moreira et al. [14]	2022	Mixed	Psychologists implemented cognitive–behavioral therapy and digital tools to support mental health during the pandemic.
	Valdez et al. [15]	2021	Mixed	The pandemic increased anxiety, irritability, and emotional regressions among individuals with ASD and their families.
	Amorim et al. [17]	2020	Mixed	Confinement heightened anxiety and adaptation difficulties in individuals with ASD.
Behavioral and Psychopathological Impacts	De la Cruz [18]	2022	Quantitative	The emotional and psychopathological impacts included increased anxiety, irritability, and repetitive behaviors; caregiver support was critical.
	Soubeste et al. [19]	2023	Mixed	Social isolation led to higher anxiety, aggression, and disruptive behaviors due to therapy interruptions.
	Zhao et al. [22]	2021	Qualitative	Anxiety sensitivity was identified as a mediator between autistic traits and PTSD symptoms related to the pandemic.
Effects of Isolation and the Disruption of Routines on Behaviors and the Emotional State	Mosquera et al. [24]	2021	Qualitative	Isolation reduced social pressure but increased loneliness and vulnerability in individuals with ASD.
	Lugo et al. [25]	2021	Mixed	Confinement increased anxiety and altered the perception of daily functioning in individuals with ASD.
	Núñez et al. [26]	2021	Mixed	Confinement intensified behavioral difficulties linked to family stress and psychosocial factors.
Coping and Adaptation Strategies	Prieto et al. [21]	2022	Quantitative	Family interventions and personalized therapies were crucial to mitigate emotional effects and foster adaptation.
	Coelho et al. [16]	2022	Qualitative	Family coexistence improved communication and autonomy, benefiting emotional stability.
Impacts on Mental Health and Behavior	Levante et al. [27]	2021	Mixed	Families of individuals with ASD experienced higher stress levels compared to neurotypical families during confinement.
	Martínez et al. [28]	2021	Quantitative	Significant increases in anxiety and repetitive behaviors were observed in individuals with ASD compared to neurotypicals.
	Vibert et al. [23]	2023	Quantitative	Continuity of services contributed to emotional stability and symptom management during and after the pandemic.
	Stadheim et al. [20]	2022	Qualitative	Isolation caused social regressions and increased disruptive behaviors due to the loss of routines and professional support.
